# Redefining the treponemal history through pre-Columbian genomes from Brazil

**DOI:** 10.1038/s41586-023-06965-x

**Published:** 2024-01-24

**Authors:** Kerttu Majander, Marta Pla-Díaz, Louis du Plessis, Natasha Arora, Jose Filippini, Luis Pezo-Lanfranco, Sabine Eggers, Fernando González-Candelas, Verena J. Schuenemann

**Affiliations:** 1 Institute of Evolutionary Medicine, University of Zurich, Zurich, Switzerland; 2 Department of Evolutionary Anthropology, University of Vienna, Vienna, Austria; 3 Department of Environmental Sciences, University of Basel, Basel, Switzerland; 4 Unidad Mixta Infección y Salud Pública, FISABIO/Universidad de Valencia-I2SysBio, Valencia, Spain; 5 CIBER in Epidemiology and Public Health, Instituto de Salud Carlos III, Madrid, Spain; 6 Department of Biosystems Science and Engineering, ETH Zürich, Basel, Switzerland; 7 Swiss Institute of Bioinformatics, Quartier Sorge, Lausanne, Switzerland; 8 Zurich Institute of Forensic Medicine, University of Zurich, Zurich, Switzerland; 9 Department of Genetic and Evolutionary Biology, University of São Paulo, São Paulo, Brazil; 10 Institute of Environmental Science and Technology (ICTA) and Prehistory Department, Universitat Autònoma de Barcelona, Bellaterra, Spain; 11 Department of Anthropology, Natural History Museum Vienna, Vienna, Austria; 12 Human Evolution and Archaeological Sciences (HEAS), University of Vienna, Vienna, Austria

**Keywords:** Evolutionary biology, Evolutionary genetics, Bacterial genetics, Bacterial infection

## Abstract

The origins of treponemal diseases have long remained unknown, especially considering the sudden onset of the first syphilis epidemic in the late 15th century in Europe and its hypothesized arrival from the Americas with Columbus’ expeditions^1,2^. Recently, ancient DNA evidence has revealed various treponemal infections circulating in early modern Europe and colonial-era Mexico^3–6^. However, there has been to our knowledge no genomic evidence of treponematosis recovered from either the Americas or the Old World that can be reliably dated to the time before the first trans-Atlantic contacts. Here, we present treponemal genomes from nearly 2,000-year-old human remains from Brazil. We reconstruct four ancient genomes of a prehistoric treponemal pathogen, most closely related to the bejel-causing agent *Treponema pallidum* *endemicum*. Contradicting the modern day geographical niche of bejel in the arid regions of the world, the results call into question the previous palaeopathological characterization of treponeme subspecies and showcase their adaptive potential. A high-coverage genome is used to improve molecular clock date estimations, placing the divergence of modern *T. pallidum* subspecies firmly in pre-Columbian times. Overall, our study demonstrates the opportunities within archaeogenetics to uncover key events in pathogen evolution and emergence, paving the way to new hypotheses on the origin and spread of treponematoses.

## Abstract (web summary)

Reconstruction of four *Treponema pallidum* genomes associated with human remains from around 2,000 years ago suggests that *T. pallidum* existed in the Americas and diverged to its modern subspecies before the fifteenth century European contact with the Americas.

## Main

Treponemal infections, caused by *T. pallidum* bacteria, are increasing at alarming rates around the world^7–11^. Increasing evidence suggests that many treponemal strains have developed antibiotic resistance, which is expected to facilitate their spread^12^. This re-emerging threat has led to many modern genetic and medical studies^8,13–15^. The closely related *T. pallidum* subspecies *T. pallidum pallidum* (TPA), *T. pallidum pertenue* (TPE) and *T. pallidum endemicum* (TEN)—responsible for syphilis, yaws and bejel, respectively—have highly similar genome sequences that differ by approximately 0.03%^16,17^. Today, bejel is geographically concentrated in arid, hot environments, especially the eastern Mediterranean and western Asia, whereas yaws is mainly found in the humid, warm tropics such as Africa or South America^18^. Among treponematoses, syphilis is the most globally distributed; it is widespread even in wealthy Western populations with easy access to health care^7,13^. By contrast, yaws and bejel mostly affect developing countries and remain less extensively studied^18^.

Historically, venereal syphilis is known for having caused a devastating outbreak in Europe in the late 1400s. Symptoms that may develop in the absence of effective treatment include severe physical disfigurement, blindness and mental impairment^19^. As similar manifestations can appear in all treponematoses^20–22^, their distinction at the subspecies level is often unreliable and mostly based on the location of characteristic skin ulcers (on the genitals or elsewhere), especially in developing countries with limited medical resources^23–25^. Diagnoses from historical cases are similarly difficult: although treponematoses can leave pathological alterations in bones, these appear in only approximately 5–30% of advanced cases^26,27^, resulting in probable underestimation of the past prevalence of treponematoses.

The early presence and potential origin of syphilis in Europe was proposed in the pre-Columbian hypothesis, based on osteological analyses of treponemal lesions, whereas the Columbian hypothesis associates its emergence with Columbus’ first American expedition and considers the contradicting palaeopathological evidence to be unreliable^2^. Before the distinctions among the subspecies could be genetically defined^28,29^, the unitarian hypothesis claimed that all treponematoses were the same disease, which only manifested differently under different environmental and cultural factors. Attempts to identify subspecies using palaeopathology have had ambiguous results and require DNA evidence as confirmation: previous ancient DNA studies have, for example, revealed that some cases of presumed syphilis instead correspond to yaws^4,5^, and recovered at least one previously unknown *T. pallidum* strain^4^. Since treponemes possess an impressive ability to adjust to various environments and are known to have previously occupied geographical regions outside their present distributions^4,5^, only unequivocally pre-Columbian treponemal DNA evidence can illuminate the origins of syphilis, and may also unravel important aspects of the evolutionary history of all treponemes.

Here, we present evidence of pre-Columbian treponemal disease in the New World from a nearly 2,000-year-old Brazilian sambaqui burial site, Jabuticabeira II, through four reconstructed genomes of *T. pallidum* with up to 33.6× coverage, phylogenetically basal to the modern diversity of the bejel-causing subspecies, *T. pallidum endemicum*.

## Geographical origins and palaeopathology

**Fig. 1:**
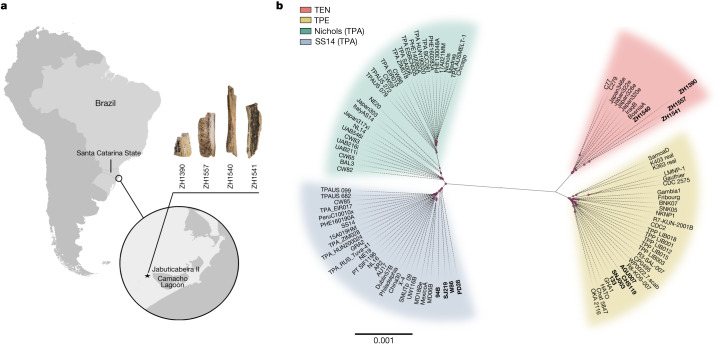
The archaeological site and the *T. pallidum*-positive samples that yielded the reconstructed genomes. **a**, A map showing the location of the Jabuticabeira II archaeological site on the south coast of Santa Catarina state, Brazil, and the samples ZH1390, ZH1540, ZH1541 and ZH1557, for which genomes were reconstructed. **b**, A maximum-likelihood phylogenetic tree of the modern and ancient *T. pallidum* strains using GTR + G + I (see Methods) as the evolutionary model and 1,000 bootstrap repetitions. All ancient genomes used in this study (newly reconstructed and previously published ancient genomes; see Supplementary Table 3) are marked in bold. Pink dots represent nodes with bootstrap values exceeding 70%. The scale bar is in units per substitutions per site.

**Extended Data Fig. 1:**
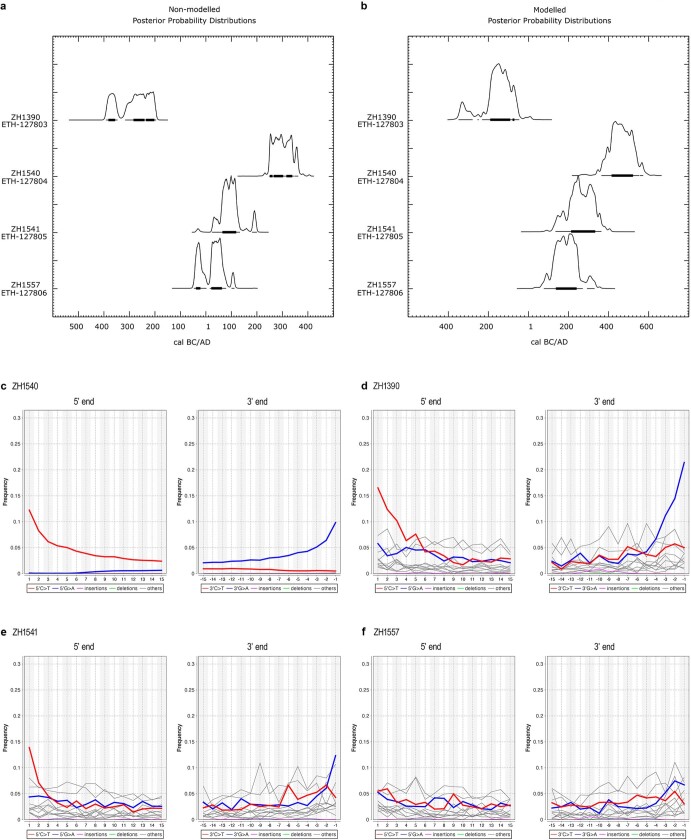
Calibrated 14C-dating curves of samples used for ancient genome reconstruction and damage profiles for aDNA authentication. Calibration conducted by Calib radiocarbon calibration program, showing the **a**) pre- and **b**) post-reservoir effect corrected (modelled) curves for the four samples (ZH1390, ZH1540, ZH141 and ZH1557) included in the genomic analyses. Damage profiles obtained with the DamageProfiler tool^130^ show the misincorporation patterns induced by age, for each sample that yielded a reconstructed genome: **c)** ZH1540, **d**) ZH1390, **e**) ZH1541 and **f**) ZH1557. A pattern of cytosine-to-thymine base misincorporation accumulating at the end of the reads is indicative of authentic ancient DNA in the sample.

Ninety-nine specimens from Jabuticabeira II from the Laguna region of Santa Catarina on the Brazilian coast, both with and without pathologies, were incorporated in this study. Previous osteological analyses had revealed infection-related pathologies suggesting potential treponemal infections^30^, such as periostitis (24 cases), bone remodelling (4 cases) and moth-eaten marks on the cranium (4 cases). Of the 37 samples considered preliminarily positive for treponemal DNA after the initial screening, 12 were from individuals with pathologies, and the rest came from non-pathological specimens (Supplementary Table 1). Four bone samples, from four different individuals, yielded sufficient genomic data for comprehensive analyses. Sample ZH1390 (Table 1 and Fig. 1a) represents a tibia fragment showing periostitis. Sample ZH1540 came from a set of commingled bones of an incomplete skeleton, namely from a fibula with pathological lesions (Table 1 and Fig. 1a). Samples ZH1541 and sample ZH1557 originated from long bones without any identified pathologies (Table 1 and Fig. 1a). All samples were radiocarbon-dated and tested for the marine reservoir effect. The raw, calibrated and corrected data from ^14^C dating are presented in Extended Data Fig. 1a,b, Supplementary Table 2 and Methods, ‘Archaeological information’. The individuals positive for treponemal DNA were not buried separately from other individuals in Jabuticabeira II, suggesting that they were treated equally.

**Table 1:** Summary of the sample information and central statistics

Index ID	Archive ID	Molecular sex (from SG data)	Raw reads	Mapped reads (post-duplicate removal)	Average coverage	1× genome coverage (%)	2× genome coverage (%)	3× genome coverage (%)	5× genome coverage (%)	SNPs
ZH1540	FS9-L3-T2	XX	19,661,672	567,158	33.60	99.71	99.65	99.57	99.38	123
ZH1390	41A-L2.05-E4	Unknown	43,980,864	83,348	2.10	9.22	9	3.63	2.31	272
ZH1541	FS3B-L3-T4	XX	99,335,748	122,086	2.67	18.45	18.32	8.66	5.74	215
ZH1557	2B-L6-E3	Unknown	88,186,424	179,285	3.92	19.41	19.25	9.74	6.70	316

Identifiers and molecular sex for the four individuals representing the samples yielding the reconstructed genomes. Statistical data on the DNA content in the samples, including the number of raw reads, reads after duplicate removal, average coverage and genomic coverage from 1× to 5× per sample, and the final number of single nucleotide polymorphisms (SNPs) covered for each. SG, shotgun sequencing.

## Preliminary pathogen screening

In the initial screening from shotgun sequencing data, 37 out of 99 samples showed between 7 and 133 hits to *Treponema* family taxa in the Kraken database, and were included in the target-enrichment process (Supplementary Table 1 and Methods, ‘Sample processing’). Of these samples, 9 had more than 5,000 reads that mapped to 3 *T. pallidum* reference genomes (BosniaA, CDC2 and Nichols) post-capture, and were thus considered positive for treponemal infection (Supplementary Table 1). For these positive samples, three additional double stranded libraries were produced for a second round of genome-wide enrichment^4,31^ (for a detailed methodology, see Methods, ‘Sample processing’). After the additional enrichment, the data from all libraries made from the same original extracts and all sequencing data produced in the two rounds of capture were combined for each sample. Four samples, ZH1390, ZH1540, ZH1541 and ZH1557, had reads covering 9.2–99.4% of the BosniaA reference genome at 1×, with an average coverage between 2× and 33.6× (Table 1). These four samples were considered as having the most potential for whole-genome reconstruction and downstream analyses.

## Authenticity estimation of ancient DNA

The authenticity of ancient DNA was confirmed by examining the deamination of bases at the ends of reads: 21%, 10%, 12% and 7% at the 5′ ends and 17%, 12%, 14% and 6% at the 3′ ends for the ZH1390, ZH1540, ZH1541 and ZH1557 samples, respectively (Extended Data Fig. 1c–f). The samples had average fragment lengths^32–34^ ranging from 64 bp to 74 bp (Table 1). Additionally, the sex chromosome assignment from the shotgun data was consistent with XX for samples ZH1540 and ZH1541. Although the individuals were previously deemed as likely males in osteological analyses, the samples ZH1390 and ZH1557 yielded insufficient data for molecular sex determination (Table 1 and  Methods, ‘Archaeological information’).

## Genome reconstruction

**Fig. 2:**
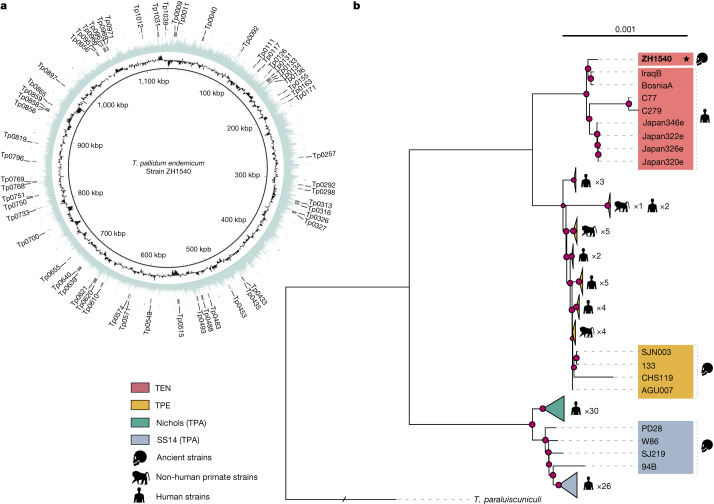
Analysis of the high-coverage genome ZH1540. **a**, Circular plot of the ZH1540 genome. Circles indicate (from inside outwards): genomic position, GC content (black) and coverage (blue). The outer rim (grey) shows a set of 60 candidate genes associated with virulence and outlined in previous studies^3,4^. **b**, Collapsed maximum-likelihood phylogenetic tree obtained using the whole-genome alignment and using *Treponema paraluiscuniculi* to root the tree using GTR + G + I (see Methods) as the evolutionary model and 1,000 bootstrap repetitions. The new ancient genome ZH1540 is highlighted in bold and with a star. Sublineages within each subspecies or lineage are collapsed, with the exception of the ancient genomes and the TEN clade. Pink dots represent nodes with bootstrap values exceeding 70%.

After high-throughput Illumina sequencing of the enriched DNA from the 4 selected samples, the resulting 20–100 million raw reads were merged sample-wise and duplicate reads were removed (Table 1). Genomes were reconstructed by mapping each sample to three representative high-quality reference genomes of *T. pallidum* subspecies: CDC2 for TPE, BosniaA for TEN, and Nichols for TPA (Methods, ‘Sample processing’ and ‘Dataset selection’). We filtered positions on the basis of read coverage, variant allele frequency, *P* value and base quality, and obtained three different consensus sequences for each sample, each with a different number of covered bases, as well as SNPs. The number of SNPs in each sequence, along with the phylogenetic analyses consistently supported a placement of all four samples within the TEN clade (Figs. 1b and 2b, Table 1 and Supplementary Table 3). Although the consensus sequences from three samples, ZH1390, ZH1541 and ZH1557, were assigned to *T. pallidum* *endemicum* (Fig. 1b), read coverage was below the threshold required for downstream analyses (for details, see Methods ‘Sample processing’ and ‘Read processing and multiple reference-based genome alignment generation’).

The final sequence obtained for the ZH1540 sample resulted in 99.38% coverage with respect to the TEN reference genome (BosniaA), a minimum coverage depth of 5× and a median depth of 33.6× (Table 1 and Fig. 2a). Variant calling resulted in the identification of 123 SNPs, each of which was checked individually (details provided in Supplementary Table 3 and in Methods, ‘Sample processing’ and ‘Read processing and multiple reference-based genome alignment generation’). Of the available modern references, the new ancient TEN genome exhibits a difference of 123 SNPs compared with the BosniaA and IraqB samples. However, the number of differing positions is much higher compared with the 4 Japanese TEN genomes (205 SNPs) and the Cuban TEN genomes (504 SNPs).

## Multiple reference-based genome alignment

The new ancient genome ZH1540 was analysed together with an additional 98 publicly available genomes, including 8 modern TEN strains, 30 TPE strains (including 9 genomes from primates and 4 ancient genomes), 30 Nichols-lineage and 30 SS14-lineage TPA strains (including 4 ancient genomes) (Supplementary Table 3). Assembly files for 33 of these 98 genomes were available and downloaded directly from the public databases European Nucleotide Archive (ENA) and National Center for Biotechnology Information (NCBI). For the remaining 65 genomes, we mapped the raw sequencing data to the closest of four representative reference genomes (CDC2, BosniaA, Nichols and SS14), to obtain new assembly files. The genome reference selected for each sample was based on the subspecies and/or lineage classification of each sample from the original publications (Supplementary Table 3). A multiple reference-based genome alignment of 98 sequences from several sources was generated according to the previously published methodology^35^. The resulting alignment spanned a total of 1,141,812 nucleotides with 6149 SNPs detected (see Data availability and Methods, ‘Sample processing’ and ‘Read processing and multiple reference-based genome alignment generation’).

## Phylogenetic and recombination analyses

A reliable phylogenetic reconstruction required the removal of non-vertically inherited genomic regions, such as recombinant regions or loci with intra- or intergenic conversion. In a recombination analysis with the phylogenetic incongruence method^36^ (PIM), we detected 34 recombinant regions across 27 genes, encompassing a total of 957 SNPs (15.56% of the total SNPs) (Supplementary Table 3 and Supplementary Table 4). Owing to the exclusion of the highly passaged Seattle-81 strain, 3 of the previously detected recombinant genes were not detected here, and 11 detected genes were novel in relation to the previously published results. The average length of the recombinant regions was 368 bp, with a minimum length of 4 bp and a maximum of 2,209 bp. Notably, all the recombination events detected here correspond to inter-subspecies transfers with the exception of an intra-subspecies recombination event found in the *tp0117* gene and three additional genes for which the putative donors are unidentified external sources (Supplementary Table 4 and Methods, ‘Recombination analysis using PIM’ and ‘PIM procedure for likelihood mapping and topology tests’).

**Extended Data Fig. 2:**
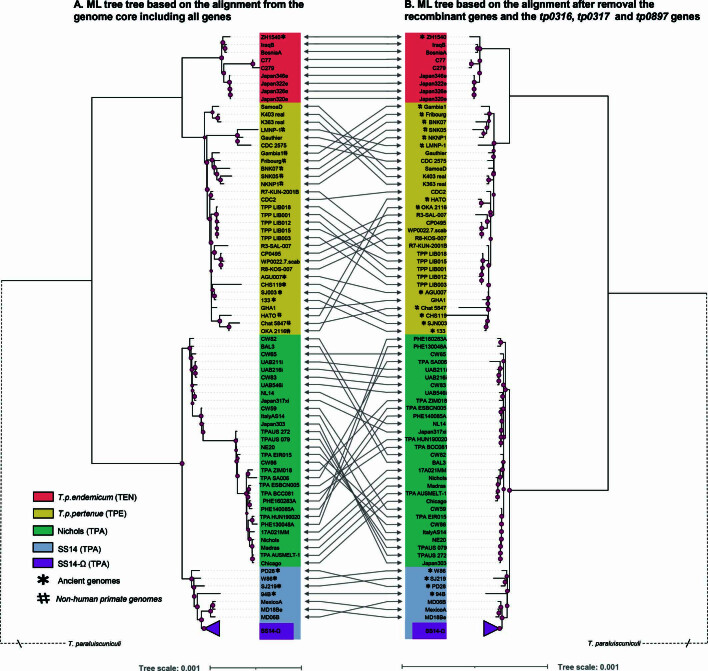
Comparison of the topologies of maximum-likelihood (ML) trees. **a**) ML tree topology with all genes included in the multiple genome alignment using GTR + G + I as the evolutionary model and 1000 bootstrap repetitions. **b**) ML tree topology after excluding *tp0897*, *tp0316* and *tp0317* and recombinant genes from the multiple genome alignment using GTR + G + I as the evolutionary model and 1000 bootstrap repetitions. The different clades corresponding to TPE and TEN, and the Nichols and SS14 lineages of TPA are colour-coded according to the legend. Bootstrap support values higher than 70% are indicated by pink circles, with circle size proportional to bootstrap support. The subclade SS14-Ω, that includes all SS14-lineage TPA strains except ancient TPA strains, MD06B, MD18Be and MexicoA, is collapsed.

**Extended Data Fig. 3:**
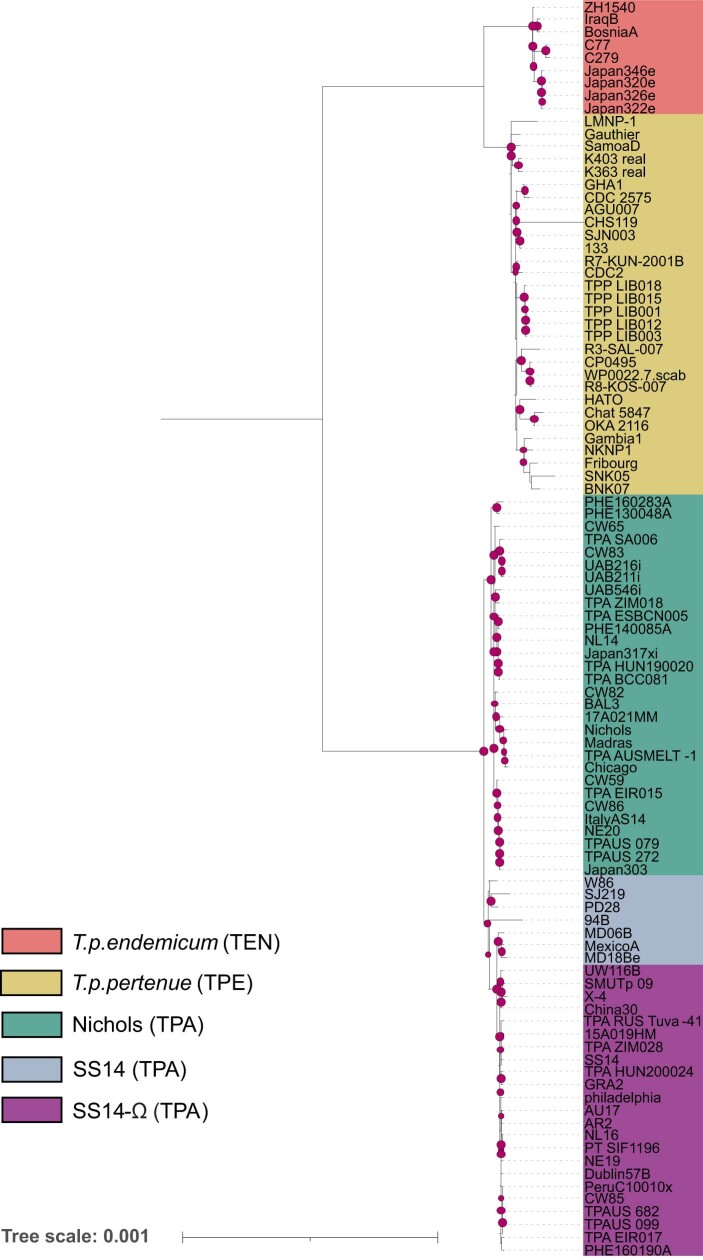
Maximum-likelihood (ML) tree with recombinant, hypervariable and 16S, 23S genes excluded. ML tree obtained after excluding *tp0897*, *tp0316, tp0317, 16* *S, 23* *S* and recombinant genes from the multiple genome alignment using GTR + G + I as the evolutionary model and 1000 bootstrap repetitions. The different clades corresponding to TPE and TEN, and the Nichols and SS14 lineages of TPA are colour-coded according to the legend. Bootstrap support values higher than 70% are indicated by pink circles, with circle size proportional to bootstrap support.

To construct a strictly vertical-inheritance alignment we removed the 27 recombinant genes detected here along with three genes, *tp0316*, *tp0317* and *tp0897*, that are known to be hypervariable and/or subject to gene conversion^37,38^, from the initial alignment (see Data availability and Methods, ‘Phylogenetic analysis’). The final recombination-free alignment spanned 1,103,436 bp with 3,718 SNPs. Maximum-likelihood trees were built using both multiple genome alignments (Fig. 2b and Extended Data Figs. 2 and 3). In Extended Data Fig. 2, the topologies of the two maximum-likelihood trees with and without the recombinant or hypervariable loci are compared.

The elimination of non-vertically inherited genes had a minor effect on the reconstruction of the *T. pallidum* phylogeny (Extended Data Figs. 2b and 3 and Methods, ‘Phylogenetic analysis’). The results after removing recombinant sites detected in PIM were confirmed with two other recombination detection programs, Gubbins and ClonalFrameML (Methods, ‘Recombination analysis using Gubbins and ClonalFrameML’). Additionally, gene mutations (A2058G and A2059G) related to macrolide antibiotic resistance were assessed^12^, and were found to be absent in the ancient genome ZH1540 (Methods, ‘Exploratory characterization of the 16S-23S genes’).

## Molecular clock dating

**Fig. 3:**
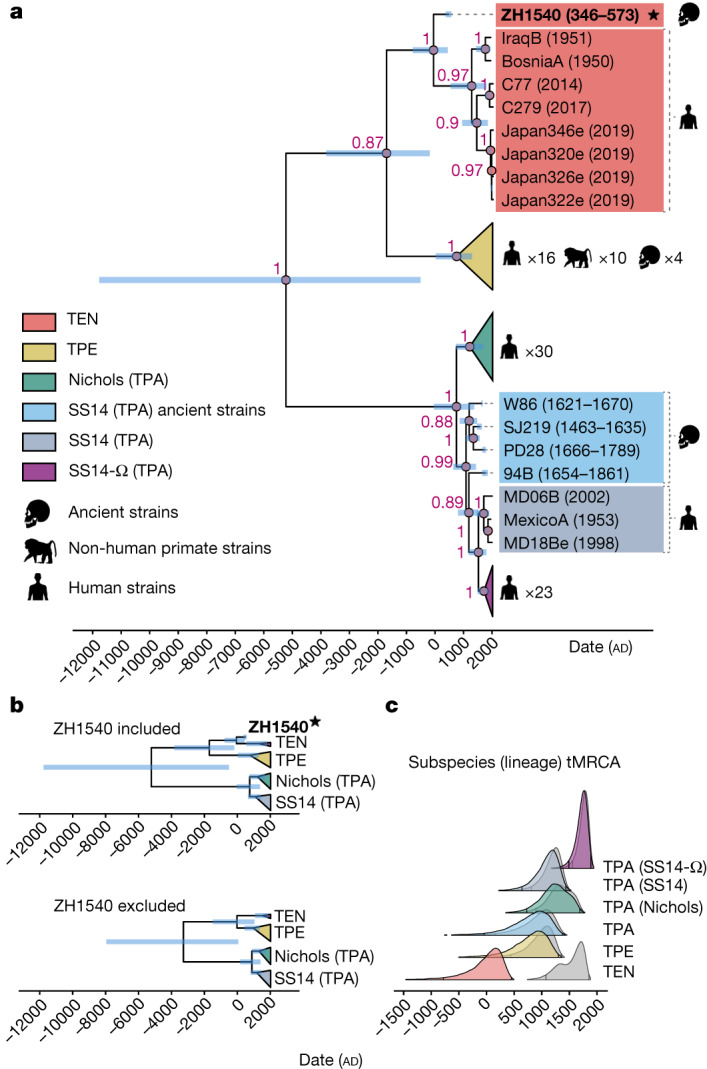
Molecular clock dating results. **a**, Maximum clade credibility (MCC) tree of previously published ancient and modern genomes, and the ancient genome ZH1540 from this study (*n* = 99). Blue bars indicate the 95% HPD intervals of node ages and red text the posterior probability that a group is monophyletic (only shown for nodes with posterior probability >0.8). **b**, Simplified collapsed MCC tree of the analysis with ZH1540 included (top) and excluded (bottom). **c**, Posterior densities of the times of the tMRCAs of the *T. pallidum* subspecies and major lineages as estimated by the molecular clock dating with ZH1540 included (coloured densities, corresponding to **b**, top) and excluded (grey densities, corresponding to **b**, bottom). Vertical lines inside the density curves indicate the upper and lower limits of the 95% HPD intervals.

**Extended Data Fig. 4:**
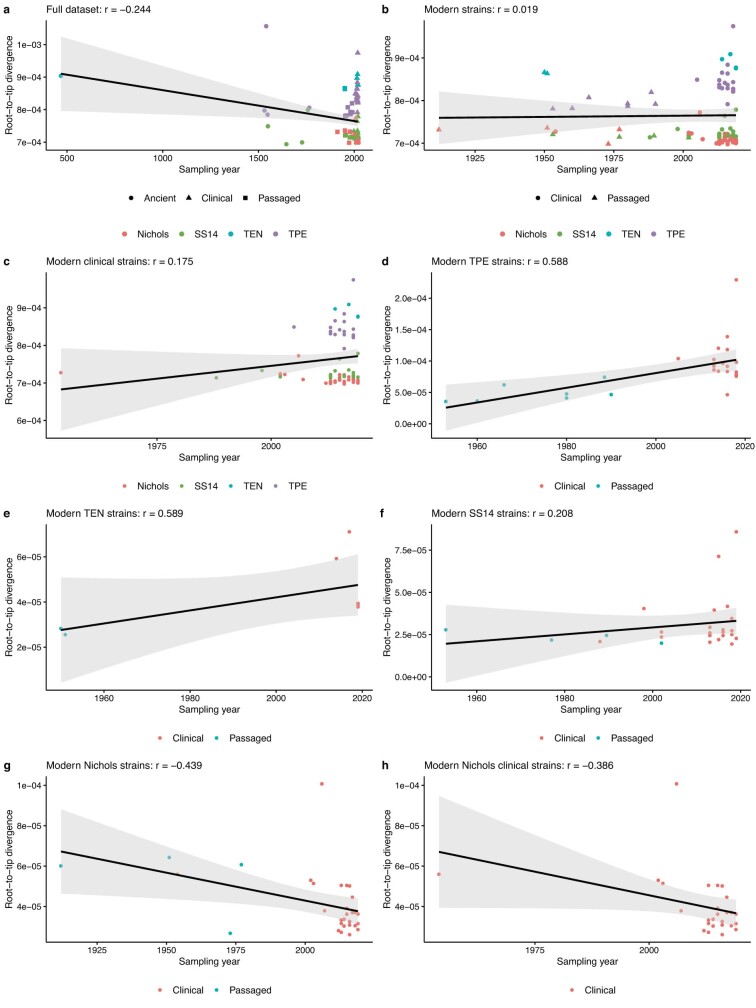
Root-to-tip divergence against sampling date of *T. pallidum* strains and respective Pearson correlation coefficients. The shaded areas denote the 95% confidence interval of the regression lines. **a**) Full dataset. **b**) Modern strains only. **c**) Modern clinical strains only. **d**) Modern TPE strains only. **e**) Modern TEN strains only. **f**) Modern TPA SS14 strains only. **g**) Modern TPA Nichols strains only. **h**) Modern TPA Nichols clinical strains only.

Molecular clock dating was performed on the same dataset as above, with 27 recombinant genes, *tp0316*, *tp0317* and *tp0897* removed. In the estimated time-calibrated phylogeny, all three subspecies (TEN, TPE and TPA), as well as the SS14 and Nichols lineages of TPA received high support for forming distinct clades (posterior probability >0.97; Fig. 3a and Extended Data Table 1). As in the maximum-likelihood phylogeny, the new ancient genome, ZH1540, occupies a basal position within the TEN clade, with all modern TEN strains forming a monophyletic subgroup (posterior probability 0.96; Fig. 3a). The majority of SS14 strains fall within the previously defined SS14-Ω subclade^4,16^, which also receives high posterior support. According to the results of root-to-tip regression analyses (Extended Data Fig. 4), an uncorrelated log-normally distributed (UCLD) and an uncorrelated exponentially distributed (UCED) relaxed-clock model were chosen for the molecular clock dating analysis, both with a narrow lognormal prior with a mean (in real space) of 1 × 10^−7^ substitutions per site per year and s.d. of 0.25 on the mean clock rate. Consistent with the previously reported results of molecular clock dating^4,35^, we find that all historical TPA strains fall basal to all modern SS14 strains, and together these form a well-supported clade (posterior probability 0.97). We therefore consider the historical strains to fall within the SS14 clade.

**Extended Data Fig. 5:**
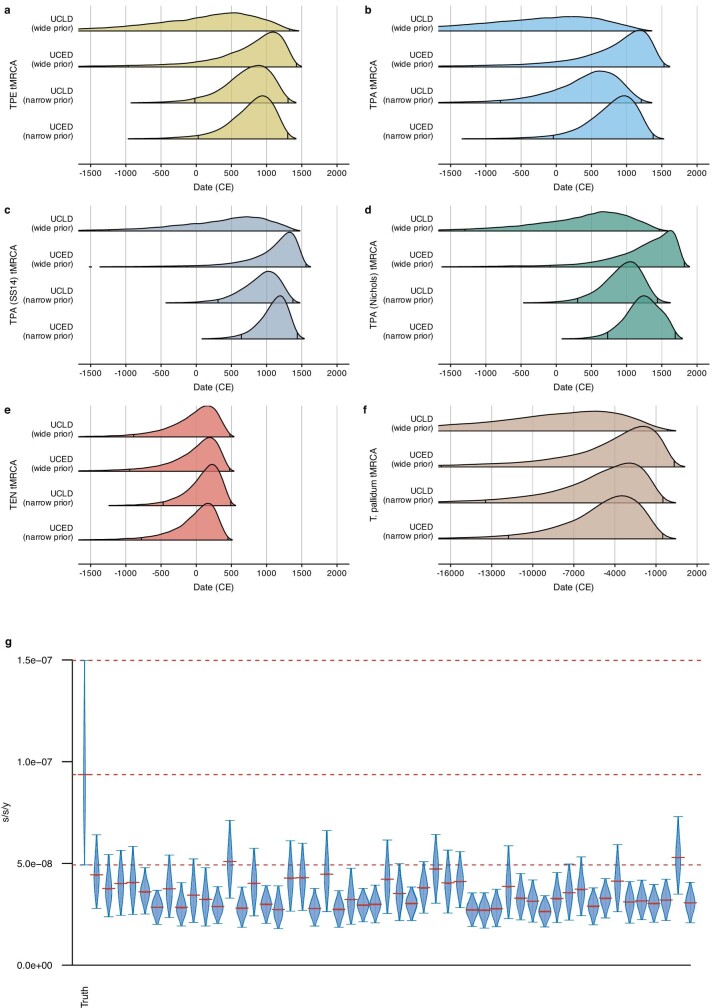
Posterior densities of the times of the most recent ancestors (tMRCAs) of the *T. pallidum* subspecies and major lineages as estimated by the molecular clock dating under different relaxed clock models and the results of the Bayesian date randomization test (DRT). **a**) TPE, **b**) TPA, **c**) TPA SS14, **d**) TPA Nichols, **e**) TEN and **f**) *T. pallidum*. Vertical lines inside the density curves in panels a-f indicate the upper and lower limits of the 95% HPD intervals. **g**) Posterior distributions for the mean clock rate, using the true, unpermuted sampling dates (far left) and 50 replicates with sampling dates permuted among tips under a UCED model with a narrow mean clock-rate prior. Distributions are truncated at the upper and lower limits of the 95% HPD intervals and horizontal red lines indicate the median estimates. The red dashed lines indicate the median and the limits of the 95% HPD interval of the mean clock rate estimated under the true sampling dates.

**Extended Data Fig. 6:**
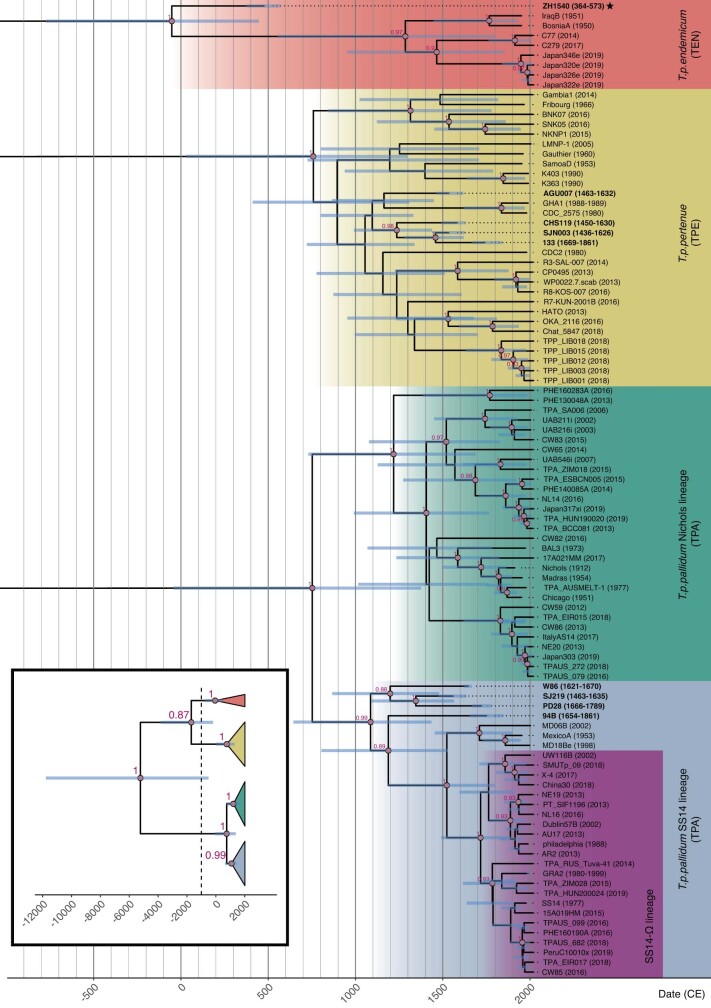
Maximum-clade credibility (MCC) tree from the molecular clock dating analysis of the 98 genome context dataset with ZH1540 included (n = 99). The inset shows a simplified view of the entire tree, with the dashed line indicating the part of the tree shown in the main figure. Ancient genomes are labelled in bold text and ZH1540 is marked by a star. Blue bars indicate the 95% HPD intervals of node ages and red text the posterior probability that a clade is monophyletic (only shown for nodes with posterior probability > 0.8).

**Extended Data Fig. 7:**
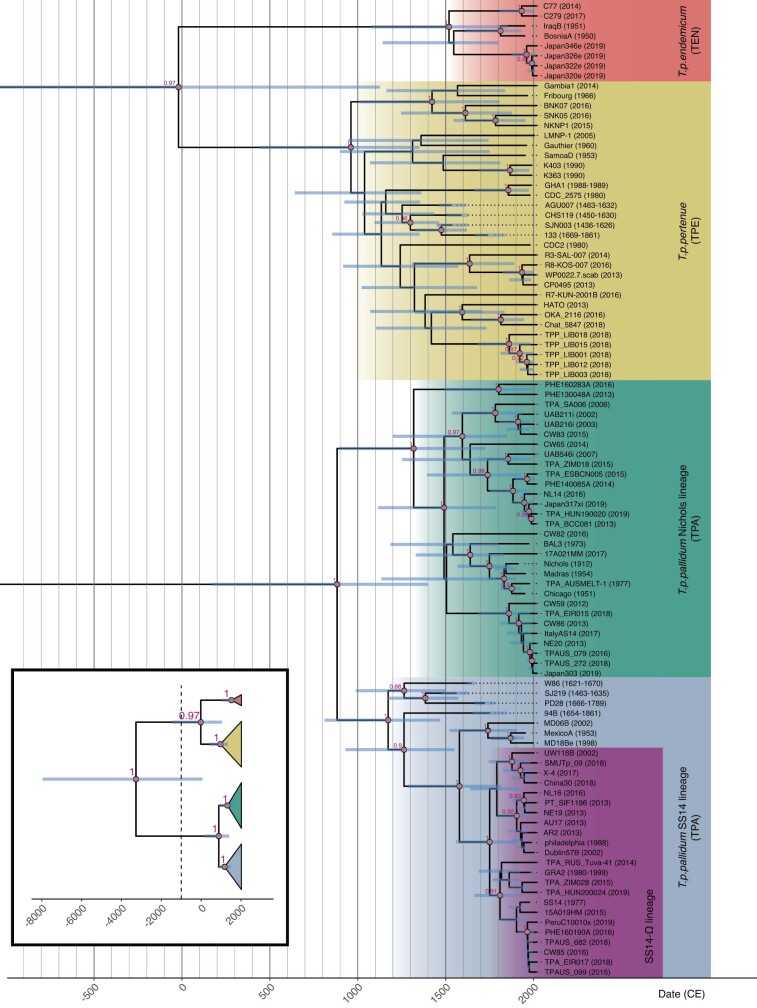
Maximum-clade credibility (MCC) tree from the molecular clock dating analysis of the 98 genome context dataset with ZH1540 excluded (n = 98). The inset shows a simplified view of the entire tree, with the dashed line indicating the part of the tree shown in the main figure. Ancient genomes are labelled in bold text. Blue bars indicate the 95% HPD intervals of node ages and red text the posterior probability that a clade is monophyletic (only shown for nodes with posterior probability > 0.8).

The age of ZH1540, which is parametrized by the radiocarbon dating results, acts as a constraint on the time of the most recent common ancestor (tMRCA) of the TEN clade. The inclusion of one far older sample results in earlier divergence times, with wider credible intervals for all major clades in the tree (Fig. 3b and Extended Data Figs. 5–7). This effect is most pronounced for TEN, where the 95% highest posterior density (HPD) interval of the tMRCA stretches from 780 bc to 449 ad (236–1845 ad for the subclade comprising only modern TEN strains), but is limited to 1077–1855 ad when excluding ZH1540 (Extended Data Figs. 5–7 and Extended Data Table 1). For all other major lineages, the effect is more moderate, and while the lower limit of the 95% HPD interval can be several hundred years older when including ZH1540 (around 400 years in the case of TPE), the upper limit is never much more than 50 years older (Fig. 3c, Extended Data Table 1 and Extended Data Figs. 6 and 7).

Although the median estimates of lineage divergence times are older than those reported previously (TEN, 47 ad; TPE, 835 ad; TPA, 844 ad; Nichols, 1238 ad; SS14, 1127 ad; and SS14-Ω, 1738 ad), the 95% HPD intervals largely overlap with estimates reported elsewhere^5,35^ (Extended Data Table 1). The two exceptions are TEN and SS14-Ω, which we estimate to have a possibly much older origin than previously thought, regardless of whether ZH1540 is included. This is probably owing to the more diverse dataset used here, which more accurately represents the full genetic diversity of the SS14-Ω lineage. Similarly, when including ZH1540 the overall *T. pallidum* tMRCA is estimated as much older than previously estimated^4,5,35^.

We caution that although we performed a relaxed-clock analysis, we did not explicitly model lineage-specific or time-dependent substitution rates. Both phenomena could explain the older age of the TPA lineages estimated here compared to previous studies, and time-dependent rates could also push the subspecies and overall *T. pallidum* tMRCAs even further into the past. As such, the results presented here should be interpreted as lower bounds on the divergence times of *T. pallidum* clades, leaving open the possibility of estimating older divergence times with the recovery of more high-quality ancient genomes and the development of improved molecular clock models.

## Discussion

Many previous hypotheses—relying solely on palaeopathological evidence—have suggested early treponemal infections among the prehistoric populations in the Americas^2,30,39^. Here we present ancient DNA evidence of a pre-Columbian New World treponematosis by reconstructing a high-coverage *T. pallidum* genome retrieved from nearly 2,000-year-old Brazilian indigenous human remains, along with three low-coverage genomes from the same spatiotemporal context. Unexpectedly, these genomes are remarkably similar to those of the causative agent of modern day bejel, *T. pallidum endemicum*. As syphilis has been the central focus of *Treponema* research, the endemic treponematoses have received less attention^2,40^. Contrary to yaws and syphilis, both of which have been previously found in Old and New World contexts from the early modern period^3–6^, this newly reconstructed genome represents the first TEN-like pathogen isolated from archaeological remains, and the only ancient comparison to the current set of eight published bejel genomes^17,38,41,42^. Recent cases have shown that treponematoses are occasionally transmitted in an atypical manner for their genetically confirmed subspecies, and have challenged their geographical and symptom-driven categorizations^43–45^. Our findings in this study only enforce this view: an ancient, TEN-like agent, identified far from the disease’s modern day geographical niche, in a humid Brazilian coastal region attests to the ability of treponemes to adapt to various climates and geographic locations. Excluding the bone damage observed in some of the studied remains, the clinical symptoms, severity and evolutionary history of the newly found TEN-like ancient pathogen remain unknown. Indeed, discoveries of other ancient pathogens, such as the early Eurasian presence of plague from the late Neolithic onwards^46^, and *Salmonella paratyphi* C, with the possible connection to the major *cocoliztli* epidemic in the mid-16th century Mexico, have shown that historically devastating diseases may have represented unexpected serovars and display a much-altered distribution today^47–50^. High-quality treponemal DNA recovered from a prehistoric source validates the use of ancient DNA techniques in establishing an entirely novel, more informed hypothesis on the events leading to the spread of *Treponema pallidum* across the world.

### Bejel in focus

In the present day, the neglected tropical disease bejel is mainly found in arid African, western Asian and Mediterranean regions, making it an unlikely candidate for a potential South American treponematosis from a coastal context^23^. Although genetically unconfirmed, palaeopathological cases of potential treponematoses found worldwide^2,51–53^ may indicate that bejel was previously more widespread and possibly associated with different environmental habitats. Our genomic investigation, together with the radiocarbon dating of both human remains and stratigraphy, places the newly found treponematosis in South America long before the European contact in the 15th century, even predating the Viking expeditions to the North American coast—firmly attesting to the presence of bejel-like treponemal infections in the pre-contact New World. Phylogenetically, this prehistoric form belongs indisputably to the TEN clade, basal to all of its modern strains. Overall, the TEN genomes are highly similar to each other, which may indicate a slow evolution of the lineage as a whole, at least until recently. Regardless of the improved genomic representation of the modern TEN genomes and the newly reconstructed pre-Columbian genome in this study, a larger representation of this lineage would be needed to draw robust conclusions about the evolution and diversification of the subspecies.

### Consequences for *T. pallidum* evolution

The data presented here include one exceptionally high-quality and high-coverage ancient treponemal genome^4–6,35^, and push back the dates for the oldest reconstructed ancient *T. pallidum* strains by more than 1,000 years. Although the ancient DNA fragments recovered in this study were not adequate for de novo assembly or pan-genome analyses, the high coverage obtained enabled us to conduct a detailed analysis at the gene and SNP level. Our findings reveal numerous inter-subspecies recombination events, which are known to be a key mechanism in bacterial evolution that result, for example, in the acquisition of virulence factors or other adaptive traits. Since the recombination events identified here involve both ancient and modern strains, at least one of the endemic forms remained in geographical proximity and in a common host pool with the TPA strains after their initial divergence. When and where exactly these recombination events took place is unknown. However, the divergence of clades can be estimated via molecular clock dating for the different branches of the *T. pallidum* phylogenetic tree. The calibration of this method is based on the known ages of the utilized genomes, making securely radiocarbon-dated ancient genomes indispensable to the analysis. Our new high-coverage ancient genome provides an unprecedented, prehistoric calibration point for molecular clock dating, and enables us to conclude that all three subspecies had already diverged from each other before Columbus’ voyages. The new estimates for the tMRCA of all *T. pallidum* (12,006–545 bc) and the emergences of the modern clades (TEN: 780 bc–449 ad, TPE: 28–1299 ad and TPA: 42 bc–1376 ad) are much earlier than previous estimates that relied on modern and previously published historical genomes. Yet, these are only the lower bounds of the divergence times, and the subspecies could have originated even earlier. Only genetically ancestral forms of treponemes could illuminate whether the early American strains spread with the early human dispersals—some 15,000–23,000 years ago^29,54^—or resulted from a local, perhaps zoonotic event. Finally, as the breakthrough discovery of a pre-Columbian treponematosis here is the result of a combination of ancient pathogen genomics and the careful selection of archaeological samples, we can expect future findings to illuminate the events leading to the rise and spread of venereal syphilis, and help resolve the evolutionary factors responsible for the global success of the *Treponema* family.

## Methods

### Inclusion and ethics

Genetic studies of ancient human diseases shed light on how past populations thrived and dealt with health problems, which may trigger concerns such as stigmatization due to diseases or rights and legal issues among people living today. Historical injustices, colonization, and dispossession have often complicated indigenous communities’ ability to assert and maintain their territorial rights in a legal or administrative framework. It is therefore crucial to consider, besides the scientific aspects, also the perspectives of living (indigenous) communities and people when carrying out this work^55^.

Here we study human remains of fully anonymous individuals who died more than 1,000 years ago and were buried in the archaeological site Jabuticabeira II, in the municipality of Tubarao in Santa Catarina state, Brazil. This site was excavated by P. de Blasis and team^56^, funded by Fundação de Amparo à Pesquisa do Estado de São Paulo (FAPESP). and a research permit was obtained from the Brazilian National Institute of Historical and Artistic Heritage (IPHAN), according to the correspondence 1793/2019 GAB PRESI-IPHAN of the process 01506.000720/2019-65 by K. Santos Bogia. The use of the samples of the remains for this study has been also approved by P. de Blasis, custodian of the Jabuticabeira II collections at the Museum of Archeology and Ethnology of the University of Sao Paulo. The human remains have been curated, studied and sampled by S.E. and team at the University of Sao Paulo until 2016 and thereafter at the Natural History Museum of Vienna.

The territories and sites spanning across Rio Grande do Sul, Santa Catarina and Paraná are inherent to the ancestral heritage of the Kaingang, Guarani and Xokleng communities (also referred to as the ‘Sun people’ or ‘coastal group’), who are still living in the region today. These societies have not only utilized the region for mobilization and migration in search of food supply, but also traditionally traversed significant distances, leaving a trail of cultural imprints, particularly in the domain of funerary practices. In a previous study^57^, samples of five of the individuals exhumed at Jabuticabeira II were studied, revealing some genetic affinity with the Kaingang (a Ge-speaking group of Southern Brazil). However, to the best of our knowledge, the Kaingang are not seen as direct descendants of sambaqui societies, nor do they identify with the people who once dwelled at Jabuticabeira II or request their remains. Finally, research in the Instituto Socioambiental (https://www.socioambiental.org/; for the defence of Brazilian socio-environmental diversity, including Indigenous Rights) states that the region around Jabuticabeira II is not part of any Indigenous reserve, nor are there claims of groups for territorial rights of this region or for the archaeological remains of this site (P. de Blasis, personal communication).

Degenerative processes, often resulting from contexts of marginalization, conflict and displacement, bear witness to the impact of historical relationships of the indigenous groups with colonizers and invaders. The afflictions and diseases experienced by these groups carry historical and environmental ramifications of notable significance, warranting explicit acknowledgment and examination. Regarding possible stigmatization of local (indigenous) communities and persons affected with bejel, it must be stressed that this contagious disease is an endemic, mostly non-sexually transmitted disease, common in hot regions where people live in close contact to each other, have no need for especially covering clothing, and share utensils. Today bejel, which can lead to stigmatization owing to disfiguring wounds, occurs especially in east-mediterranean and west-African communities with limited access to modern medical care. Although the World Health Organization realizes the importance of actions taken to eradicate bejel worldwide since 1949 (WHA2.36 Bejel and other Treponematoses (https://www.who.int/publications/i/item/wha2.36)), the disease is not seen as a current public health issue in Brazil, as it is for some other countries^58,59^. This stands in contrast to the high prevalences of sexually transmitted diseases such as HIV and venereal syphilis, which affect the indigenous communities in Brazil (Em São Paulo, ação em aldeias promove debate e testagem rápida de HIV e Sífilis — Fundação Nacional dos Povos Indígenas (http://www.gov.br/funai/pt-br/assuntos/noticias/2019/em-sao-paulo-acao-em-aldeias-promove-debate-e-testagem-rapida-de-hiv-e-sifilis)). It is, however, notable that, in the archaeological context, nothing implied that those prehistoric people of Jabuticabeira II with the local treponematosis would have been discriminated against in their time and culture.

Furthermore, the culturally insensitive descriptions in palaeogenomic research articles are an ethical issue of concern^60^. To ensure discretion, we curated for potentially insensitive or discriminatory expressions within the manuscript. Importantly, we had the invaluable help of E. Krenak, Cultural Survival’s lead on Brazil, Indigenous activist and PhD candidate at the University of Vienna, to critically analyse our texts and provide advice in ethically correct and fair use of terminology.

### Archaeological information

#### The sambaquis of the Laguna region

A sambaqui is the prevalent type of archaeological site on the Brazilian coast: a human-built shell midden or shell mound of varying dimensions, located in rich resource areas such as lagoons, mangroves or estuaries. Sambaquis consist of inorganic sediment, mollusc shells, food debris and organic matter mixed in intricate stratigraphies associated with domestic and/or funerary functions^61^. More than 1,000 sambaquis are mapped along the 7,500 km long Brazilian coast and are dated to between 7,500 and 1,000 yr bp ^61,62^. Recent archaeological research suggests that these shell mound-building populations were sedentary, with an abundant and stable marine-based subsistence, horticulture, high population growth^61,63^, elaborate funerary rituals^64^ and landscape appropriation^56^.

#### Jabuticabeira II excavation site

Jabuticabeira II (UTM 22 J − 0699479E; 6835488 S) is a medium-sized shell mound (400 × 250 × 10 m in height), settled on a palaeodune and located in the Laguna region, the highest density area of sambaquis from the southern Brazilian coast, 3 km from Laguna do Camacho, one of several water sources associated with a barrier-lagoon geological system formed during the Holocene (Fig. 1). Jabuticabeira II, built during a nearly 1,000-year period, is one of 65 sambaquis mapped around the lagoon system. This large number of settlements and their chronologically overlapping occupation history attest to a fairly dense occupation and intense interactions of the sambaqui builders between 7,500 and 900 calibrated years (cal yr) bp ^56^. According to stratigraphic studies, Jabuticabeira II is the result of incremental funerary rituals accumulated over centuries. Although Jabuticabeira II was not completely excavated, 204 burials containing the remains of 282 individuals were exhumed from a 373 m^2^ area^64^. Radiocarbon dates of Jabuticabeira II stratigraphy^56,64^ suggest a long occupation period between 1214–830 cal bc and 118–413 cal ad or 3137–2794 to 1860–1524 cal yr bp (2σ), roughly in line with the radiocarbon datings from bone material of the four individuals in this study, ranging from 350 cal bc to 573 cal ad.

The human remains from the Jabuticabeira II sambaqui were found in single, double, and multiple burials, dispersed in clusters. The skeletons recovered were mostly incomplete, avoiding categorical estimations of age and sex or other osteological findings. The burial pattern was tightly flexed and suggested intentional treatment of the body prior to the internment. The small size of the graves suggested that the bodies suffered previous desiccation or decomposition of soft tissues, but not enough to produce complete disarticulation (hand and feet bones were found articulated). Many burials come from profiles and are incomplete. The bones of several individuals are stained with red ochre^65^, a common practice in archaeological sites of the Santa Catarina state^66,67^. Offerings are common in sambaqui burial contexts and include adornments made with faunal material and lithic tools in a wide range of forms, from debris to polished tools and zooliths, with differences in frequency of occurrence among different sites and strata^68^. The most common offering in Jabuticabeira II was fish.

Altogether, 99 Jabuticabeira II individuals, with and without bone alterations suggestive of infection, were screened for pathogen DNA content. 37 samples deemed positive for treponemal DNA in the initial screening and four samples yielded sufficient data for *T. pallidum* genome reconstruction (Supplementary Table 1).

#### Palaeopathological analysis of treponematoses

Bioarchaeological analyses showed results compatible with increasing population growth and high population density in Jabuticabeira II, including high frequencies of nonspecific stress markers^69^ and occasional infant stress^70^, but no evidence of trauma associated with interpersonal conflicts over resources or territory^69^.

There is, however, evidence of communicable systemic diseases in Jabuticabeira II and other local Brazilian sambaquis^30^. Eleven ^14^C accelerator mass spectrometry dates obtained directly from the presumably treponematosis-affected individuals suggest that these diseases are very old on the east coast of South America, with a time-range between 6,300 and 500 yr BP. Among the possible treponemal cases based on osteological analysis, three came from Jabuticabeira II. However, these did not overlap with the individuals yielding the detected genetic evidence in this study.

#### Information on individuals

##### Individual 41A-L2.05-E4, sample ZH1390

The individual is an adult male of robust build, with an estimated stature of 150.49 ± 2.6 cm (ref. ^70^). Although fragmented, the bones of this individual comprised an almost complete skeleton (80%), articulated and buried in an oval shell-rich matrix in a hyper-flexed position. The bones of the individual showed signs of systemic infectious disease in the lower limbs. Femurs, tibias, and fibulas all show discrete generalized periostitis and osteoarthrosis. A widening in the lateral portion of clavicles was also observed. According to Filippini et al.^30^, applying the SPIRAL method^71^, this individual’s disease could be classified non-conclusively as syphilis, yaws or bejel. The sampling was performed on an active lesion on the tibia fragment.

##### Individual FS9–L3–T2, sample ZH1540

The sample comes from an assemblage of commingled bones, of probably more than one individual. The bones assigned to this individual consist of several skeletal elements, some with pathological alterations, such as severe osteomyelitis in the distal third of the right humerus, severe periostitis in the left ulna, periostitis in a fibula diaphysis, and two vertebral bodies with osteophytosis. The sample was taken from the fibula fragment, in the area with periostitis.

##### Individual FS3B-L3-T4, sample ZH1541

The sample comes from one of three separate individuals, found commingled. The skeletal elements belonging to this robust adult of unknown age and sex include a left radius with arthritis, a fragment of the left ulna (very robust), a fragment of the left humerus, fragments of a femur, a tibia, and a fibula and a first metatarsal. The sample was taken from a femur fragment, under the immediate surface of the bone, to best avoid the possible introduction of external contaminants.

##### Individual 2B-L6-E3, ZH1557

The sample comes from a probably adult male individual. The individual was articulated and in a flexed position with another, adult female individual buried on top. Osteopathological findings on the bones of the sampled individual included signs of degenerative joint disease, severe lumbar intervertebral osteoarthritis, scoliosis, and possible injuries to the patellae. However, no typical lesions suggestive of treponemal infection were observed. The sample was taken from a small piece of long bone, under the immediate surface of the bone, to best avoid the possible introduction of external contaminants.

#### Marine reservoir effect correction for ^14^C dating

Radiocarbon dating was performed by the Laboratory of Ion Beam Physics at ETH Zurich (laboratory number: ETH-127328) using bone collagen purified by a modified ultrafiltration method^72^. Data calibration was done with OxCal v4.4.4. The diet of the Jabuticabeira II inhabitants, substantially consisting of marine food sources, produces a reservoir effect in the radiocarbon dates calculated as mean age of 247.8 (*σ* = 103.7) years^73^. Considering the high contribution of marine carbon to bone collagen of individuals in Jabuticabeira II, the radiocarbon dates were modelled with Calib Rev 8.20^74^ (http://calib.org/calib/calib.htm) using the Mixed Marine SHCal20 calibration curve^75,76^ and applying the estimated average local marine radiocarbon reservoir correction value (Δ*R*) of −126 ± 29 for the South coast of Brazil (Marine Reservoir Correction database)^73,77^. We considered the average relative contribution of marine carbon to collagen derived from Bayesian Mixing Models for Jabuticabeira II individuals, calculated at a mean value of 42.5%^78,79^. For the individual estimates for the samples, see Supplementary Table 2.

#### Sample processing

Samples were documented and carried through sampling, DNA extraction, library preparation and library indexing in facilities dedicated to ancient DNA work at the University of Zurich, including decontamination of samples, laboratory equipment and reagents with UV irradiation and using protective clothing and minimum contamination-risk working methods.

All post-amplification steps were performed in the regular laboratory facility available for the Paleogenetics Group at the Institute of Evolutionary Medicine (IEM), University of Zurich (UZH). DNA sequencing was performed at the Next Generation Sequencing facility of the Vienna BioCenter Core Facilities (VBCF) or at the Functional Genomics Center at the University of Zurich (FGCZ).

#### Ancient DNA extraction

All sample surfaces were irradiated with ultraviolet light to minimize potential contamination from modern DNA. The bone powder was obtained using a dental drill and diamond head drill bits. DNA extraction was performed on around 50–100 mg of bone powder, according to a well-established extraction protocol for ancient DNA^80^. Negative controls for extraction and library processes were processed in parallel through all experiments, one control per ten samples, sequenced and bioinformatically compared to their corresponding sample batches, as precaution against possible contamination.

#### Library preparation

Double stranded DNA libraries were produced for initial screening with shotgun sequencing, without UDG treatment (that is, chemical treatment aiming to limit age-related damage in the DNA). Two additional libraries for each of the potentially positive samples from the first round of capture were produced to maximize the DNA complexity. For the preparation of DNA libraries, 20 µl of DNA extract was converted into double stranded DNA libraries^31^. Sample-specific barcodes (indexes) were added to both ends of the DNA fragments in the libraries^81^. The indexed libraries were then amplified to reach a minimum DNA concentration of approximately 90 ng ml^−1^. The amplification was performed using 1× Herculase II buffer, 0.4 mM IS5 and 0.4 mM IS6 primer^81^, Herculase II Fusion DNA Polymerase (Agilent Technologies), 0.25 mM dNTPs (100 mM; 25 mM each dNTP) and 5 ml indexed library as DNA template. Four reactions per library were prepared and the total amplification reaction volume was 100 ml. The thermal profile included an initial denaturation for 2 min at 95 °C and 3–18 cycles, depending on DNA concentration after indexing of the libraries, denaturation for 30 s at 95 °C, 30 s annealing at 60 °C and a 30 s elongation at 72 °C, followed by a final elongation step for 5 min at 72 °C. All splits of one indexed library were pooled and purified using the QIAGEN MinElute PCR purification kit. DNA libraries were then quantified with D1000 ScreenTape on an Agilent 2200 TapeStation (Agilent Technologies) and combined in equimolar pools for sequencing.

#### Pathogen screening

Shotgun data were used for an initial screening of the 99 candidate samples, with Kraken2 software^82^, and 41 samples that had more than 7 hits to *T. pallidum* were selected for target enrichment. The samples selected were subjected to a target enrichment process and subsequently processed by FastQ Screen v0.15.1^83^ to check the number of mapped reads against three representative high-quality reference genomes of *T. pallidum* subspecies (CDC2, BosniaA and Nichols). The nine most promising samples (>5,000 Kraken hits to *T. pallidum* after first round of in-solution capture), were turned into two extra libraries and re-captured as explained in detail in the following sections.

#### Target enrichment for *T. pallidum* DNA

Genome-wide enrichment of double stranded libraries was performed through custom target enrichment kits (Arbor Bioscience). RNA baits with a length of 60 nucleotides and a 4 bp tiling density were designed based on three reference genomes: Nichols (CP004010.2), SS14 (CP000805.1), Fribourg-Blanc (CP003902). 500 ng library pools were enriched according to the manufacturer’s instructions. Captured libraries were amplified in 100 µl reactions containing 1 unit Herculase II Fusion DNA polymerase (Agilent), 1× Herculase II reaction buffer, 0.25 mM dNTPs, 0.4 mM primers IS5 and IS6^81^ and 15 µl library template, with the following thermal profile: initial denaturation at 95 °C for 2 min, 14 cycles of denaturation at 95 °C for 30 s, annealing at 60 °C for 30 s, and elongation at 72 °C for 30 s, followed by a final elongation at 72 °C for 5 min. Captured libraries were purified with MinElute spin columns (QIAGEN) and quantified with a D1000 High Sensitivity ScreenTape on an Agilent 2200 TapeStation.

#### Sequencing

For both shotgun data retrieval and after the capture processing, the samples were pooled in unimolar quantity (for SG sequencing up to 50 samples per pool, and for the capture process 2–8 samples per pool), and sequenced on an Illumina NextSeq500 with 2 × 75  +  8  +  8 cycles using the manufacturer’s protocols for multiplex sequencing at the Functional Genomics Center in Zurich or at the Vienna BioCenter Core Facilities.

### Statistical analyses

#### Dataset selection

We assembled a genomic dataset comprising 98 publicly available *T. pallidum* genomes (8 TEN, 30 TPE and 60 TPA) from previously published studies (including 8 ancient genomes), and the newly generated ZH1540 genome. The genomes represent the genetic variation of the three known subspecies of *T. pallidum* (TPA, TPE and TEN) available by December 2022, and were selected with a focus on TEN and TPE, because of their proximity to the new ancient genome classified as TEN.

Published data for the modern genome dataset in this study are available at the European Nucleotide Archive (ENA) database: PRJNA313497 (accession numbers: SRR3268682, SRR3268724, SRR3268715, SRR3268694, SRR3268696, SRR3268709, SRR3268710), PRJEB11481 (accession numbers: ERR1470343, ERR3596780, ERR3596747, ERR3596783), PRJEB28546 (accession numbers: ERR4045394, ERR3684452, ERR3684456, ERR3684465, SRR13721290, ERR4853530, ERR4993349, ERR4853587, ERR4899206, ERR5207017, ERR5207018, ERR5207019, ERR4899215, ERR4853623, ERR4853625), PRJNA508872 (accession numbers: SRR8501165, SRR8501164, SRR8501167, SRR8501166, SRR8501168, SRR8501171), PRJNA723099 (accession numbers: SRR14277267, SRR14277266, SRR14277458, SRR14277444), PRJEB11481 (accession number: ERR1470331), PRJDB9408 (accession numbers: DRR213712, DRR213718), PRJNA588802 (accession numbers: SRR10430858, SRS5636328), PRJNA322283 (accession number: SRR3584843), PRJNA754263 (accession numbers: SRR15440297, SRR15440150, SRR15440451, SRR15440240), PRJEB40752 (accession numbers: ERR4690809, ERR4690806, ERR4690810, ERR4690812, ERR4690811). Assembly files were used for 9 genomes from National Center for Biotechnology Information (NCBI) database: CP002375.1, CP002376.1, NC_016842.1, NC_017268.1, NC_018722.1, NC_021490.2, NC_021508.1, GCA_000813285.1, CP035193.1 and for 24 modern genomes from the European Nucleotide Archive (ENA): CP021113.1, CP073572.1, CP073557.1, CP073553.1, CP073536.1, CP073526.1, CP073490.1, CP073487.1, CP073470.1, CP073447.1, CP073446.1, CP073399.1, CP040555.1, LT986433.1, LT986434.1, CP032303.1, CP020366.1, CP024088.1, CP024089.1, CP078121.1, CP078090.1, CP081507.1, CP051889.1 and CP003902 .1. Raw sequence data (fastq files) used for 6 modern genomes is available at the NCBI database: PRJEB20795 (accession numbers: ERS1724928, ERS1724930, ERS1884567) and PRJNA343706 (accession numbers: SRR4308604, SRR4308606, SRR4308597). Previously published ancient treponemal genomes here used are available at the ENA: PRJEB37490 (accession number: ERR4065503), PRJEB37633 (accession number: ERR4000645), PRJEB35855, PRJEB21276 (accession numbers: ERS2470995, ERS2470994) and PRJEB62102. Detailed source information for the reference dataset is documented in Supplementary Table 3.

We selected all eight publicly available TEN genomes, all of which have more than 99.4% genome coverage, with the exception of C77^17^ (81.4%). We selected 30 TPE genomes (Supplementary Table 3). To represent each lineage or sublineage, we selected at least one genome, preferring the ones with the highest sequencing depth and genome coverage. All included TPE genomes have more than 95.3% genome coverage, except the four ancient TPE genomes: SJN003, AGU007, 133 and CHS119, displaying 97.4%, 92.7%, 57% and 62% genome coverage, respectively. Furthermore, 60 TPA genomes from the major lineages and sublineages described in previous studies were included (Supplementary Table 3). All of these genomes had more than 90% coverage, except the four ancient genomes, PD28, W86, SJ219 and 94B, all of which have genome coverage of 30% or more. All genomes in the dataset are separated from each other by at least 5 SNPs. The TPA strain Seattle-81 was excluded from the final dataset owing to mutations probably accumulated during extensive passaging in rabbits that can cause ambiguous placement in phylogenies^4,16,36^.

The raw data and/or assembly files for each genome in our dataset were downloaded from the public databases: European Nucleotide Archive (ENA)^84^ and National Center for Biotechnology Information (NCBI)^85^. Accession numbers are given in Supplementary Table 3.

#### Read processing and multiple reference-based genome alignment generation

To reconstruct the individual genomes from the raw data, we carried out raw read quality control and preprocessing, removing duplicates, variant calling and filtering using the default parameters when not otherwise specified. After processing the de-multiplexed sequencing reads, sample sequencing quality was analysed with FastQC version 0.11.9^83^, filtering reads with a QC value < 25. Following processing by cutadapt version 4.1^86^ to remove the sequencing adapters, in order to reduce the reference bias, and improve the posterior phylogenetic inference and assignment^87^, the genome reference selection for mapping each sample was determined according to the results from the original manuscript where the genomes were published (see Supplementary Table 3). The mapping was carried out by BWA mem^88^ (using parameters: -k 19, -r 2.5). Four reference genomes were used; the well-studied TEN and TPE genomes BosniaA (NZ_CP007548.1) and CDC2 (NC_016848.1), as well as the Nichols (NC_021490.2) and SS14 (NC_010741.1) genomes, representing the two main lineages within TPA. However, for the new ancient samples obtained here, genomes for each sample were reconstructed by mapping to three high-quality reference genomes, representing the three *T. pallidum* subspecies (CDC2, BosniaA and Nichols).

CleanSam, from Picard Toolkit version 2.18.29 (http://broadinstitute.github.io/picard), was used to clean the provided SAM or BAM files. Duplicate reads were removed using MarkDuplicates, from Picard toolkit version 2.18.29. AddOrReplaceReadGroups, from Picard Toolkit version 2.18.29, was used to assign all the reads in a file to a single new read-group before using mapDamage version 2.2.0-86-g81d0aca^89^ to estimate the DNA damage parameters and rescale quality scores of probably damaged positions in the reads (using parameter: --rescale).

After generating a text pileup output for the BAM files with the mpileup tool from Samtools version 1.7^90^, SNPs were called using VarScan version 2.4.3^91^ (using parameters: -p-value 0.01, -min-reads2 1, -min-coverage 1, -min-freq-for-hom, 0.4 -min-var-freq 0.05, -output-vcf 1). Next, a SNP filtering was also carried out with VarScan (using for the modern samples parameters: -p-value 0.01, -min-reads2, 5 -min-coverage 10, -min-avg-qual 30 -min-freq-for-hom 0.4, -min-var-freq 0.9, -output-vcf 1; and modifying some parameters for the ancient samples because of their lower read coverage and quality: -p-value 0.01 -min-reads2 3, -min-coverage 5, -min-avg-qual 30, -min-freq-for-hom 0.4, -min-var-freq 0.9 -output-vcf 1). Additionally, all positions with less than 3 mapped reads were masked with Genomecov from Bedtools version 2.26.0^92^ for modern and ancient samples. All steps of genome generation were visualized and manually confirmed with Tablet version 1.21.02.08^93^, checking each SNP one by one and discarding the possible spurious SNPs from the new ancient genome ZH1540. The resulting final sequences were obtained by maskfasta from Bedtools v2.26.0.

Additionally, we used tested sequencing and posterior analysis methodologies^17,42^ to obtain higher coverage and more reliable modern *T. pallidum* genomes. Where possible, assembly files were obtained rather than raw data (Supplementary Table 3). A multiple reference-based genome alignment for all sequences was generated in MAFFT v7.467^94^ (using parameters: --adjustdirection --auto --fastaout --reorder). However, due to the use of different genomic references, regions with low coverage for some genomes, corresponding mostly to *tpr* and *arp* genes, were reviewed and manually aligned with Aliview version 1.25^95^.

The samples ZH1390, ZH1541, and ZH1557 had sufficient data to attempt a genome reconstruction and were determined to have the most SNPs in common with the TEN reference but they were excluded from downstream analyses due to the limited coverage acquired for each of them, which made the obtained SNPs less reliable. The sample ZH1540, however, yielded a remarkable 33.6× genomic coverage and was selected for subsequent in-depth analyses.

Proteinortho version 6.0b^96^ (using parameters: -p=blastn -singles -keep) was used to conduct an orthology study in order to find orthologous genes in the four reference genomes used^96^. Each gene present in at least one of the four reference genomes had its genomic coordinates determined based on its location in the final merged alignment (see Supplementary Table 3).

To verify the accuracy of the final multiple genome alignment, and that no protein-coding gene was inadvertently truncated, the protein translations for every gene present in at least one reference genome were compared to the original gff3 files of each of the four references (Supplementary Table 3). The reconstructed ZH1540 genome and its main features were represented graphically using BRIG version 0.95-dev.0003^97^.

#### Recombination analysis using PIM

As previously noted^36^, the presence of recombination in the genomes of *T. pallidum* may interfere with the topologies of phylogenetic trees inferred. In order to look into potential gene recombination, we used the PIM pipeline^36^ to detect recombination gene by gene. In brief, the process involved the following steps:

1. Using IQ-TREE version 1.6.10, a maximum-likelihood tree was created for the multiple genome alignment^98^. All maximum-likelihood trees for the remaining steps were obtained using GTR^99^ + G^100^ + I^101^ as an evolutionary model and 1,000 bootstraps replications.
2. The 1,161 genes found in at least one of the reference genomes were extracted, and the number of SNPs for each gene was calculated. Genes with less than three SNPs were excluded.
3. The phylogenetic signal in each gene alignment for each of the remaining genes was evaluated by likelihood mapping^102^ in IQ-TREE (using parameters: -lmap 10000 -n 0), retaining only those genes that showed a phylogenetic signal.
4. A maximum-likelihood tree was generated for each of the remaining genes using IQ-TREE.
5. For each included gene, we tested the phylogenetic congruence between trees using IQ-TREE (using parameters: -m GTR + G8 -zb 10000 -zw), comparing the maximum-likelihood tree obtained from the gene alignment and the maximum-likelihood tree obtained from the whole-genome alignment using two different methods: Shimodaira–Hasegawa^103^ and expected likelihood weights (ELW)^104^. Genes for which at least one test rejected the reference tree topology with the gene alignment adopting a conservative approach (*P* < 0.2, weight value close to 0, for Shimodaira–Hasegawa and ELW tests, respectively) and the complete genome alignment rejected the topology of the tree built using the gene alignment (reciprocal incongruence, *P* < 0.2 and weight value close to 0) in at least one of them were selected and examined more closely in the next step.
6. Using MEGAX^105^, the selected genes that displayed reciprocal incongruence were subsequently examined to assess and describe potential recombination events. A gene has to have at least three nearby homoplastic SNPs—SNPs that are shared by several groups (TPE, TEN, TPA-Nichols or TPA-SS14) and produce a polyphyletic distribution—in order to be labelled as recombinant. The homoplastic SNPs found in the gene alignment served as the boundaries of the recombinant areas.
7. Using a parsimony criterion on the distribution of alternative states of the homoplastic SNPs, the potential donor and recipient clades or strains of each recombination event were inferred.

DNA sections, a number of genes have a high percentage of sites with missing data. The majority of these genes are members of the *tpr* and *arp* families, which include collections of paralogous genes. In order to continue analysing these intriguing genes with the PIM pipeline, strains that had a high percentage of missing data in each of these genes were eliminated. Following previous findings^35,36^, the hypervariable gene *tprK* (*tp0897*), with seven hypervariable regions that undergo intrastrain gene conversion^17,37,106–109^, and the *tp0316* and *tp0317* genes, also under gene conversion, were completely excluded from the recombination analysis.

#### PIM procedure for likelihood mapping and topology tests

A likelihood mapping test was performed using IQ-TREE to determine which genes (Supplementary Table 4) showed a phylogenetic signal (out of the 382 genes for which >3 SNPs were found in pairwise comparison with at least one reference genome). For each quartet (subset of four sequences) in the data, the test creates unrooted phylogenetic trees. The quartet likelihoods are then plotted within a triangle, where the position denotes the ‘tree-likeness’ of the quartet in question. Corner quartets are completely resolved, quartets on the sides are partially resolved, and quartets in the centre are unresolved. Of the 382 genes, 29 had too many missing values to be tested using the likelihood mapping method. In order to include these genes in the next steps of the PIM pipeline and topology comparisons, the problematic sequences with more than 50% of positions with missing data were removed.

Following the likelihood mapping test, 9 genes falling within the central zone of the triangle were discarded (Supplementary Table 4). Then, using the Shimodaira–Hasegawa and ELW topology tests, we compared the gene trees of the remaining genes to the preliminary reference tree of the whole-genome alignment to assess their phylogenetic congruence (Supplementary Table 4). Of the 373 genes that tested positive for phylogenetic incongruence, 27 contained at least three consecutive SNPs, supporting a recombination event. To these we added *tp0859*, which was detected as recombinant in a previous study^35^, resulting in a total of 27 recombinant genes.

#### Recombination analysis using Gubbins and ClonalFrameML

Gubbins version 2.3.1^110^ and ClonalFrameML version 1.11-1^111^ are frequently used tools for the genome-wide identification of recombinant positions in bacterial genomes. To test the robustness of our recombination analysis using PIM, we also ran these two programs, with default parameters and the same whole-genome alignment used with PIM. Gubbins identified 301 distinct recombination events associated with 103 genes, ranging in size from 5 bp to 13,866 bp. Similarly, ClonalFrameML detected 656 events, with 32 of them being 1 or 2 bp long, and the longest event spanning 782 bp. Notably, all the genes identified by PIM as having a recombinant region were also detected by both ClonalFrameML and Gubbins, except for gene *tp0558*, which was missed by ClonalFrameML but detected by Gubbins. Additionally, genes *tp0164* and *tp0179* were detected by ClonalFrameML but missed by Gubbins.

#### Phylogenetic analysis

A maximum-likelihood tree based on the alignment including all genes was constructed with IQ-TREE, using GTR + G + I as the evolutionary model and 1,000 bootstrap replications (Extended Data Fig. 2a). Next, genes identified as recombinant by PIM were removed from the multiple genome alignment. Three additional genes (*tp0897*, *tp0316*, and *tp0317*), which contain repetitive regions and have been identified as hypervariable and/or under gene conversion in the past, were also removed to prevent the introduction of a potential bias. Because the *tp0317* gene is nested inside the *tp0316* gene and the coordinates from the BosniaA reference genome for *tp0316* covered a larger area than those of the other reference genomes, *tp0316* and *tp0317* were removed according to the *tp0316* coordinates from the BosniaA reference genome. A reference phylogenetic tree was then constructed employing the new vertical-inheritance genome alignment, also with IQ-TREE using GTR + G + I as the evolutionary model and 1,000 bootstrap replications (Extended Data Fig. 2b). Both trees obtained were compared and are shown in Extended Data Fig. 2.

The SS14 lineage was previously described as a largely epidemic, macrolide-resistant cluster that emerged after, and was possibly prompted by, the clinical use of antibiotics following its discovery^12,16^. Based on our phylogenetic analysis results and expanding on earlier phylogenetic classifications and nomenclature of the SS14 lineage^12,16^, we defined the clade that contains almost all SS14 genomes from clinical and contemporary samples as the SS14-Ω sublineage. However, two contemporary clinical samples (MD18Be and MD06B), were not classified as SS14-Ω sublineage, because these samples cluster together with the MexicoA genome, in line with previously published results^42^.

To compare the PIM-based analysis with other widely used recombination detection methods, Gubbins and ClonalFrameML, we followed a similar procedure of removing the recombinant positions detected by these tools and inferred maximum-likelihood trees with the retained positions in the corresponding multiple genome alignments. All the phylogenetic trees with recombination events removed exhibit general congruence with each other, whether the events were identified by PIM, Gubbins or ClonalFrameML. Furthermore, the placement of the ZH1540 genome remained consistent in the phylogenetic trees, regardless of the recombination detection method employed, and despite the elimination of recombinant genes to generate the vertically inherited alignment.

#### Exploratory characterization of the 16S-23S genes

*T. pallidum* contains two rRNA (*rrn*) operons, each of which encodes the 16S-23S-5S rRNA genes and intergenic spacer regions (ISRs). There is evidence that the random distribution of *rrn* spacer patterns in *T. pallidum* may be generated by reciprocal translocation of *rrn* operons mediated by a recBCD-like system found in the intergenic spacer regions (ISRs)^112^. In concordance with previous studies^112–115^, we found that the 16S–23 S ISRs of the TPA strains contain the tRNA-Ile (tRNA-Ile-1; *tp0012*) and tRNA-Ala (tRNA-Ala-3; *tp00t15*) genes within the *rrn1* and *rrn2* operons, respectively. By contrast, the TPE genomes show an Ala/Ile spacer pattern, where the *tp0012* and *tp00t15* orthologues are located within the *rrn2* and *rrn1* operons, respectively.

We identified 68 SNPs in genes *r0001*, *r0002*, *r0004* and *r0005*, encoding the 16S-23S rRNA genes of the new ancient genome ZH1540, placing them among the most variable genes in our alignment and raising the potential that including them in the alignment could result in a biased phylogenetic reconstruction. Although the SNPs found appear to be well supported by the reads obtained from the sequence mapping (Supplementary Table 3), their origin from possible contamination cannot be completely ruled out and further analyses would be necessary to confirm them.

Excluding these genes from the alignment, in addition to the recombinant genes and *tp0316, tp0317* and *tp0897*, did not result in any changes to the topology (Extended Data Figs. 2b and 3), although branch lengths were altered. As these genes are known to have conserved regions in addition to variable regions used to explore the evolutionary relationships among pathogenic bacteria^116–118^, we decided to retain them in the alignment for all subsequent analyses. Finally, we note that the ZH1540 genome did not possess either of the two *T. pallidum* 23 S ribosomal RNA gene mutations known to confer macrolide resistance (A2058G and A2069G). In contrast, four modern TEN strains from Japan possess the A2048G mutation, suggesting recent selection pressure for antibiotic resistance mutations.

#### Molecular clock dating

We used the Bayesian phylogenetics package BEAST2 v2.6.7^119^ to estimate a time-calibrated phylogeny of the context dataset of 98 *T. pallidum* genomes along with our new ancient genome, ZH1540. We removed hypervariable and recombining genes from the alignment, as described above, reduced it to variable sites and used an ascertainment bias correction to account for constant sites.

Root-to-tip regression analyses (Extended Data Fig. 4) show that while there is a positive correlation between sampling year and root-to-tip divergence among all modern clinical strains, indicating clock-like evolution, the correlation is very weak when also including passaged strains and negative when including ancient strains. Within the TPE, TEN and SS14 clades there exists a positive correlation among all modern clinical and passaged strains. On the other hand, the correlation is negative for Nichols strains, even when looking only at clinical strains. In order to account for rate variation and the long terminal branches on some strains (likely due to a multitude of effects, including sequencing errors, contamination and mutations introduced during rabbit passaging) we used a UCLD and a UCED clock model for the molecular clock dating analysis^120^. For both models we placed a narrow lognormal prior with a mean (in real space) of 1 × 10^−7^ substitutions per site per year and standard deviation 0.25 on the mean clock rate. This strong prior was used to compensate for the poor temporal signal among *T. pallidum* genomes and was calibrated on previous estimates of the substitution rate^4,35^. We further used a GTR + G + I substitution model^118^ and a Bayesian skyline plot^121^ demographic model (tree-prior) with 10 groups. For all genomes where the sampling dates are not known exactly, we used uniform priors across the date ranges reported in the original studies to account for the uncertainty^4–6,16,122^. For ZH1540 we set the date range to 364–573 ad, in accordance with the marine reservoir effect corrected radiocarbon dating results above. Default priors were used for all other model parameters. The same analysis was repeated without ZH1540 in order to assess the effect of our new ancient genome on the divergence dates. We further repeated the analysis using a wide lognormal prior with a mean (in real space) of 1 × 10^−7^ substitutions per site per year and standard deviation 1 on the mean clock rate and using both constant-size and exponential growth coalescent models to assess the impacts of the mean clock rate prior and demographic models on divergence time estimates.

For each analysis we ran four Markov chain Monte Carlo (MCMC) chains of 5 × 10^8^ steps each, sampling parameters and trees every 10,000 steps. After assessing convergence in Tracer v1.7^123^ and confirming that all four chains converged to the same posterior distribution, we combined the chains after discarding the first 10% of samples as burn-in. In the resulting combined chains all parameters have effective sample size (ESS) values > 150. TreeAnnotator v2.6.7 was used to compute MCC trees and the results were visualized using ggplot2^124^, ggtree^125^ and custom scripts. The 95% HPD of the coefficient of variation estimated under the UCLD model excluded 0 (median = 1.46, 95% HPD 1.08–1.9), indicating that a strict clock model is not appropriate for our dataset. Robustness analyses show that under a narrow mean clock rate prior both the UCED and UCLD clock models result in similar divergence time estimates (Extended Data Fig. 5a–f), with the UCED model estimates tending to be more recent and the UCLD model estimates usually having longer tails. Under a wide mean clock rate prior, estimates with the UCED are broadly similar, albeit wider, while the UCLD model estimates very wide posterior distributions for divergence times, indicating little information under this model. Divergence time estimates were not sensitive to the demographic model used. The MCC trees under the UCED model with a narrow prior, both with and without ZH1540 included in the analysis are shown in Extended Data Figs. 6 and 7, respectively.

Finally, we performed a Bayesian date randomization test^126–128^ (DRT) to further assess the strength of the temporal signal in our dataset, by permuting sampling dates among genomes and performing 50 replicate analyses. For the analyses, the full dataset, a UCED clock model with a narrow prior and the Bayesian skyline plot demographic model were used, while fixing the sampling dates of ancient strains to the means of the radiocarbon date ranges for simplicity. MCMC chains were run for 1 × 10^8^ steps, sampling parameters every 10,000 steps. Convergence was assessed using the coda^129^ package to ensure that all parameters in all chains have ESS values > 150. The DRT results show that the 95% HPD intervals of the mean clock rate on replicates with permuted sampling dates are much smaller than expected if all information came from the mean clock rate prior (Extended Data Fig. 5g). In general, the HPD intervals do not overlap with the 95% HPD interval of the mean clock rate estimated with the true sampling dates.

### Reporting summary

Further information on research design is available in the Nature Portfolio Reporting Summary linked to this article.

## Online content

Any methods, additional references, Nature Portfolio reporting summaries, source data, extended data, supplementary information, acknowledgements, peer review information; details of author contributions and competing interests; and statements of data and code availability are available at 10.1038/s41586-023-06965-x.

## Supplementary information

Reporting Summary

Supplementary Table 1 Shotgun and post target-enrichment screening data.

Supplementary Table 2 Radiocarbon dating results for the four samples used for genome reconstruction.

Supplementary Table 3 SNP data.

Supplementary Table 4 Recombination data.

## Figures and Tables

**Extended Data Table 1:**
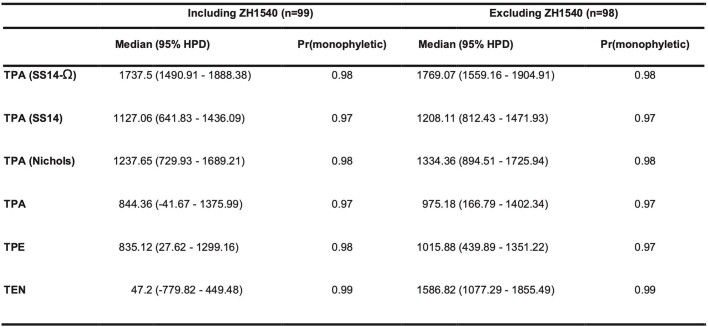
Divergence dates

Times of the most recent common ancestors (tMRCAs, in years CE) and monophyly statistics for the *T. pallidum* subspecies and major lineages within TPA, as estimated by molecular clock dating.

## Data Availability

The raw sequencing data for the four newly reconstructed ancient genomes are accessible at the European Nucleotide Archive under accession number PRJEB62647 (ERP147759). Detailed source information for the reference dataset is documented in Supplementary Table 3. The multiple reference-based genome alignment, with and without recombining regions removed, along with tree and log files for the main results and all raw data and scripts needed to reproduce analyses for this study are available at https://github.com/laduplessis/Pre-Columbian-Treponema-pallidum-from-Brazil (10.5281/zenodo.10063176).

## Code availability

No specialized in-house code was used for this study. All software used for the data analyses in this study is publicly available and cited in the main text and the supplementary information. Custom scripts and the pipeline used in the molecular clock dating analyses and visualization are deposited in https://github.com/laduplessis/Pre-Columbian-Treponema-pallidum-from-Brazil (10.5281/zenodo.10063176).
