# Supplementary material for: Redefining the treponemal history through pre-Columbian genomes from Brazil
Source: Nature. 2024 Jan 24;627(8002):182–8. doi: 10.1038/s41586-023-06965-x (PMC10917687; doi:10.1038/s41586-023-06965-x)
Supplement: Supplementary file 1 — Reporting Summary [file 41586_2023_6965_MOESM1_ESM.pdf]

nature portfolio

Corresponding author(s): Verena Schuenemann, Kerttu Majander, Fernando Gonzalez-Candelas

Last updated by author(s): Nov 1, 2023

Reporting Summary

Nature Portfolio wishes to improve the reproducibility of the work that we publish. This form provides structure for consistency and transparency in reporting. For further information on Nature Portfolio policies, see our Editorial Policies and the Editorial Policy Checklist.

Statistics

For all statistical analyses, confirm that the following items are present in the figure legend, table legend, main text, or Methods section.

n/a	Confirmed	
☐	☒	The exact sample size (*n*) for each experimental group/condition, given as a discrete number and unit of measurement
☐	☒	A statement on whether measurements were taken from distinct samples or whether the same sample was measured repeatedly
☒	☐	The statistical test(s) used AND whether they are one- or two-sided<br>*Only common tests should be described solely by name; describe more complex techniques in the Methods section.*
☒	☐	A description of all covariates tested
☒	☐	A description of any assumptions or corrections, such as tests of normality and adjustment for multiple comparisons
☐	☒	A full description of the statistical parameters including central tendency (e.g. means) or other basic estimates (e.g. regression coefficient) AND variation (e.g. standard deviation) or associated estimates of uncertainty (e.g. confidence intervals)
☒	☐	For null hypothesis testing, the test statistic (e.g. *F*, *t*, *r*) with confidence intervals, effect sizes, degrees of freedom and *P* value noted<br>*Give P values as exact values whenever suitable.*
☐	☒	For Bayesian analysis, information on the choice of priors and Markov chain Monte Carlo settings
☒	☐	For hierarchical and complex designs, identification of the appropriate level for tests and full reporting of outcomes
☐	☒	Estimates of effect sizes (e.g. Cohen's *d*, Pearson's *r*), indicating how they were calculated

Our web collection on statistics for biologists contains articles on many of the points above.

Software and code

Policy information about availability of computer code

Data collection	No specialised custom code was used. All data in this study was collected with publicly available tools. The raw data and assembly files for genome selection in our dataset were downloaded from the public databases: European Nucleotide Archive (ENA), and National Center for Biotechnology Information (NCBI).
Data analysis	No specialised in-house code was used for this study. All software used for the data analyses in this study is publicly available. All software used for data analysis in this study is publicly available, and cited in the main text and supplementary material. List of software and respective versions: OxCal v4.4.4, Calib Rev v8.20, Kraken2 sofware, FastQ Screen v0.15.1, FastQC v0.11.9, cutadapt v4.1., BWA mem, Picard toolkit v2.18.29, mapDamage v2.2.0-86-g81d0aca, Samtools v1.7, VarScan v2.4.3, Genomecov and maskfasta packages of Bedtools version 2.26.0, Tablet v1.21.02.08. MAFFT v7.467, Aliview v1.25, Proteinortho v6.0b, snp-sites v2.5.1, BRIG v0.95-dev.0003, IQ-TREE v1.6.10, MEGAX software, Gubbins v2.3.1, ClonalFrameML v1.11-1, Bayesian phylogenetics package BEAST2 v2.6.7, Tracer v1.7, TreeAnnotator v2.6.7, R-packages ggplot2, ggtree ggridges, beastio and coda. Custom scripts and pipeline used in the molecular clock dating analyses and visualisation are deposited in https://github.com/laduplessis/Pre-Columbian-Treponema-pallidum-from-Brazil (doi: 10.5281/zenodo.10063176).

For manuscripts utilizing custom algorithms or software that are central to the research but not yet described in published literature, software must be made available to editors and reviewers. We strongly encourage code deposition in a community repository (e.g. GitHub). See the Nature Portfolio guidelines for submitting code & software for further information.

Data

Policy information about availability of data

All manuscripts must include a data availability statement. This statement should provide the following information, where applicable:

- Accession codes, unique identifiers, or web links for publicly available datasets
- A description of any restrictions on data availability
- For clinical datasets or third party data, please ensure that the statement adheres to our policy

The raw sequencing data for the four newly reconstructed ancient genomes is accessible at the European Nucleotide Archive under accession number PRJEB62647 (ERP147759). Published data for the modern genome dataset in this study are available at the European Nucleotide Archive (ENA) database (https://www.ebi.ac.uk/ena/browser/home): PRJNA313497 (Accession numbers: SRR3268682, SRR3268724, SRR3268715, SRR3268694, SRR3268696, SRR3268709, SRR3268710), PRJEB11481 (Accession numbers: ERR1470343, ERR3596780, ERR3596747, ERR3596783), PRJEB28546 (Accession numbers: ERR4045394, ERR3684452, ERR3684456, ERR3684465, SRR13721290, ERR4853530, ERR4993349, ERR4853587, ERR4899206, ERR5207017, ERR5207018, ERR5207019, ERR4899215, ERR4853623, ERR4853625), PRJNA508872 (Accession numbers: SRR8501165, SRR8501164, SRR8501167, SRR8501166, SRR8501168, SRR8501171), PRJNA723090 (Accession numbers: SRR14277267, SRR14277266, SRR14277458, SRR14277444), PRJEB11481 (Accession number: ERR1470331), PRJDB9408 (Accession numbers: DRR213712, DRR213718), PRJNA588802 (Accession numbers: SRR10430858, SRS5636328), PRJNA322283 (Accession number: SRR3584843), PRJNA754263 (Accession numbers: SRR15440297, SRR15440150, SRR15440451, SRR15440240), PRJEB40752 (Accession numbers: ERR4690809, ERR4690806, ERR4690810, ERR4690812, ERR4690811). Assembly files were used for 9 genomes from National Center for Biotechnology Information (NCBI) database (https://www.ncbi.nlm.nih.gov/): CP002375.1, CP002376.1, NC_016842.1, NC_017268.1, NC_018722.1, NC_021490.2, NC_021508.1, GCA_000813285.1, CP035193.1 and for 24 modern genomes from the European Nucleotide Archive (ENA): CP021113.1, CP073572.1, CP073557.1, CP073553.1, CP073536.1, CP073526.1, CP073490.1, CP073487.1, CP073470.1, CP073447.1, CP073446.1, CP073399.1, CP040555.1, LT986433.1, LT986434.1, CP032303.1, CP020366.1, CP024088.1, CP024089.1, CP078121.1, CP078090.1, CP081507.1, CP051889.1 and CP003902.1. Raw sequence data (fastq files) used for 6 modern genomes is available at the National Center for Biotechnology Information (NCBI) database (https://www.ncbi.nlm.nih.gov/) PRJEB20795 (Accession numbers: ERS1724928, ERS1724930, ERS1884567) and PRJNA343706 (Accession numbers: SRR4308604, SRR4308606, SRR4308597). Previously published ancient treponemal genomes here used are available at the ENA: PRJEB37490 (Accession number: ERR4065503), PRJEB37633 (Accession number: ERR4000645), PRJEB35855, PRJEB21276 (Accession numbers: ERS2470995, ERS2470994) and PRJEB62102. Detailed source information for the reference dataset is documented in Supplementary Table 3. The multiple reference-based genome alignment, with and without recombining regions removed, along with tree and log files for the main results and all raw data and scripts needed to reproduce analyses for this study are available at https://github.com/laduplessis/Pre-Columbian-Treponema-pallidum-from-Brazil (doi: 10.5281/zenodo.10063176).

Research involving human participants, their data, or biological material

Policy information about studies with human participants or human data. See also policy information about sex, gender (identity/presentation), and sexual orientation and race, ethnicity and racism.

Reporting on sex and gender	n/a
Reporting on race, ethnicity, or other socially relevant groupings	n/a
Population characteristics	n/a
Recruitment	n/a
Ethics oversight	n/a

Note that full information on the approval of the study protocol must also be provided in the manuscript.

Field-specific reporting

Please select the one below that is the best fit for your research. If you are not sure, read the appropriate sections before making your selection.

☐ Life sciences ☐ Behavioural & social sciences ☒ Ecological, evolutionary & environmental sciences

For a reference copy of the document with all sections, see nature.com/documents/nr-reporting-summary-flat.pdf

Ecological, evolutionary & environmental sciences study design

All studies must disclose on these points even when the disclosure is negative.

Study description	Study addresses ancient DNA samples of Treponema pallidum pathogens from archaeologically excavated source material.
Research sample	Research sample encompasses a collection of 99 bone specimens from archaeological excavation in Santa Catarina, coastal Brazil. Four bone samples are found positive for Treponema pallidum, one of which yielded a complete ancient pathogen genome and is here investigated further with various analytic methods.
Sampling strategy	Samples were included according to a previous archaeological and palaeopathological analyses, which stated that physical signs of

Sampling strategy	infectious disease were observed in the skeletons of the individuals from the site. As palaeopathological lesions are not a secure way to ensure the presence of Treponema pallidum, we extended the archaeogenetic analyses to both samples with and without lesions. One of the four samples deemed positive for the bacterium showed visual marks of infection, whereas 3/4 were initially free of characteristic lesions.
Data collection	Data for this study was collected via high-throughput sequencing on a Illumina platform. We also conducted target enrichment for optimal amplification of the target organism's DNA in between sequencing rounds. Previously published genomic data from publicly available databases was used as a modern reference panel to ancient pathogen genomes here constructed.
Timing and spatial scale	The data was collected within two years (2020-2022), by laboratory methods specifically designed for ancient DNA and subsequent bioinformatic methods to reconstruct and analyse the retrieved pathogen genomes.
Data exclusions	No data was excluded; the screening procedure and results of pathogen DNA data not resulting in reconstructed genomes was excluded from downstream analyses, but documented and described within the manuscript.
Reproducibility	The parameters and analytic models are described in detail within the manuscript, and attainable via publicly available tools and software. The entire genomic raw sequencing data produced in this study is released for common use before publication.
Randomization	The sampling was based on the availability of archaeological findings from the studied site. To avoid biases we included both samples with and without Treponema pallidum -characteristic lesions.
Blinding	All samples experienced the same procedure in both laboratory and sequencing data analysis phase, to provide an unbiased investigation throughout the study.

Did the study involve field work? ☐ Yes ☒ No

Reporting for specific materials, systems and methods

We require information from authors about some types of materials, experimental systems and methods used in many studies. Here, indicate whether each material, system or method listed is relevant to your study. If you are not sure if a list item applies to your research, read the appropriate section before selecting a response.

Materials & experimental systems

n/a	Involved in the study
☒	☐ Antibodies
☒	☐ Eukaryotic cell lines
☐	☒ Palaeontology and archaeology
☒	☐ Animals and other organisms
☒	☐ Clinical data
☒	☐ Dual use research of concern
☒	☐ Plants

Methods

n/a	Involved in the study
☒	☐ ChIP-seq
☒	☐ Flow cytometry
☒	☐ MRI-based neuroimaging

Palaeontology and Archaeology

Specimen provenance	The use of the remains in this study has been approved by the Museum of Archeology and Ethnology of the University of Sao Paulo, under the curator Assoc. Prof. Paulo DeBlasis of Projeto Camacho, custodian of the bones of Jabuticabeira II, as well as by the IPHAN, according to the correspondence 1793/2019 GAB PRESI-IPHAN of the Process 01506.000720/2019-65 by Katia Santos Bogia. The materials have been stored and allocated for this study from the Natural History Museum of Vienna, by the curator of the collection, Prof. Dr. Sabine Eggers.
Specimen deposition	The specimens are returned to the Natural History of Vienna shortly after the publication of this study.
Dating methods	The original raw radiocarbon dating was conducted at the Laboratory of Ion Beam Physics in Zurich, Switzerland and calibrated using the IntCal 20 and using OxCal 4.4 program. The marine reservoir effect corrections were conducted with Calib 8.20 program using the Mixed Marine SHCal20 calibration curve, and applying the estimated average local marine radiocarbon reservoir correction value (ΔR) of −126±29 for the South coast of Brazil. All raw data, as well as calibrations and corrections for it are presented in the manuscript's Methods, tables and Extended Data.

☒ Tick this box to confirm that the raw and calibrated dates are available in the paper or in Supplementary Information.

Ethics oversight	All human samples used in this study are over 1000 years old, fully anonymous and unassociated directly to any living group of descendants (as far as known today). Museum of Archeology and Ethnology of the University of Sao Paulo has approved this study as custodian of the bone material. Ethical issues concerning the stigmatising nature of treponemal diseases, and the indigenous groups' rights and history in the Santa Catarina region are addressed in the Inclusion and Ethics section in Methods of this manuscript.

Note that full information on the approval of the study protocol must also be provided in the manuscript.

Plants

Seed stocks	n/a
Novel plant genotypes	n/a
Authentication	n/a
